# Crossmodal learning of target-context associations: When would tactile context predict visual search?

**DOI:** 10.3758/s13414-019-01907-0

**Published:** 2019-12-16

**Authors:** Siyi Chen, Zhuanghua Shi, Xuelian Zang, Xiuna Zhu, Leonardo Assumpção, Hermann J. Müller, Thomas Geyer

**Affiliations:** 1 General and Experimental Psychology, Department of Psychology, LMU Munich, Leopoldstr 13, 80802 Munich, Germany; 2 Center for Cognition and Brain Disorders, Institute of Psychological Sciences, Hangzhou Normal University, Hangzhou, China

**Keywords:** Visual search, Contextual cueing, Statistical learning, Crossmodal, Plasticity, Tactile processing

## Abstract

It is well established that statistical learning of visual target locations in relation to constantly positioned visual distractors facilitates visual search. In the present study, we investigated whether such a *contextual-cueing* effect would also work crossmodally, from touch onto vision. Participants responded to the orientation of a visual target singleton presented among seven homogenous visual distractors. Four tactile stimuli, two to different fingers of each hand, were presented either simultaneously with or prior to the visual stimuli. The identity of the stimulated fingers provided the crossmodal context cue: in half of the trials, a given visual target location was consistently paired with a given tactile configuration. The visual stimuli were presented above the unseen fingers, ensuring spatial correspondence between vision and touch. We found no evidence of crossmodal contextual cueing when the two sets of items (tactile, visual) were presented simultaneously (Experiment 1). However, a reliable crossmodal effect emerged when the tactile distractors preceded the onset of visual stimuli 700 ms (Experiment 2). But crossmodal cueing disappeared again when, after an initial learning phase, participants flipped their hands, making the tactile distractors appear at different positions in external space while their somatotopic positions remained unchanged (Experiment 3). In all experiments, participants were unable to explicitly discriminate learned from novel multisensory arrays. These findings indicate that search-facilitating context memory can be established across vision and touch. However, in order to guide visual search, the (predictive) tactile configurations must be remapped from their initial somatotopic into a common external representational format.

People receive a great variety of sensory inputs across time and changing contexts, imposing a huge workload on the brain. However, given that perceptual input is usually not random, the brain has developed sophisticated statistical-learning mechanisms for extracting (predictive) patterns of co-occurring sensory events over space and time. ‌‌Statistical learning can facilitate perceptual processing when a learned pattern re-occurs on later occasions. The current study is set within the rapidly growing field of statistical learning, with special emphasis on crossmodal perception.

Chun and Jiang (1998) were the first to probe statistical learning of spatial relations between target and distractors in a visual search task. In their study, participants were asked to detect/localize a (left- or right-oriented) target character “T” embedded in a set of (orthogonally oriented) distractor characters “L” and discriminate the orientation of the target “T” (left vs. right). Unbeknown to observers, half of the trials contained predictive target-distractor layouts, with non-predictive random layouts presented in the other half. Chun and Jiang (1998) found that search reaction times (RTs) were faster for predictive compared to non-predictive displays, an effect they termed *contextual cueing* (see also Chun & Jiang, 1999; Shi, Zang, Jia, Geyer, & Müller, 2013; Geyer, Shi, & Müller, 2010a). Interestingly, despite this RT advantage, observers are typically unable to reliably discern predictive from non-predictive display layouts in an explicit recognition test presented after the visual search task. These findings motivated the proposal that contextual cueing is supported by an implicit spatial (long-term) memory system (see, e.g., Chun & Jiang, 1998; but see Vadillo, Konstantinidis, & Shanks, 2016, and Annac, Pointner, Khader, Müller, Zang, & Geyer, 2019, for alternative, explicit-memory accounts of contextual cueing). Thus, contextual cueing refers to the incidental acquisition, from repeatedly searching for a target within an invariant display arrangement, of a specific form of spatial long-term memory representing the target location with respect to the (more global or local) distractor context. This representation is (automatically) activated, or retrieved, when the display arrangement is re-encountered later on and so comes to guide focal attention to the target location, for instance, by prioritizing a particular (covert or overt) attentional scan path (for recent reviews, see, e.g., Goujon, Didierjean, & Thorpe, 2015; Wolfe & Horowitz, 2017; or Sisk, Remington, & Jiang, 2019).

While most studies of contextual cueing focused on the visual modality, Assumpção and collaborators went on to examine spatial context learning in the tactile modality (Assumpção, Shi, Zang, Müller, & Geyer, 2015, 2018). They devised a novel tactile search task in which four tactile stimuli were presented on a given trial, to two out of four possible fingers of each hand (no stimuli were presented to the thumbs). The singleton target was defined by a difference in the vibrotactile stimulation pattern (one of two different target patterns delivered to one finger) relative to the three (homogeneous) distractor patterns (delivered to the other fingers). Observers responded by pressing a foot pedal (the left or the right pedal) assigned to (one or the other of) the specific vibrotactile stimulation pattern(s) delivered to the target finger. In the predictive condition, the spatial arrangements of the target and distractor fingers were held constant across trials, whereas the arrangements were generated anew on each trial in the non-predictive condition. With this novel task, Assumpção et al. (2015) established a tactile contextual-cueing effect, indicating that similar statistical-learning mechanisms are at work in tactile as in visual search.

This proposal is in line with (prior) evidence of a crossmodal transfer of learned contextual cues from a visual to a haptic search task (Nabeta, Ono, & Kawahara, 2003): In Nabeta et al., participants initially encountered predictive target-distractor arrangements in a visual search task (allowing for contextual learning) and were then presented with spatially ‘identical’ arrangements in a haptic search task (to examine for a transfer of contextual cueing). And, indeed, participants exhibited a facilitation of RTs for predictive relative to non-predictive haptic target-distractor arrangements, indicative of successful transfer of acquired contextual ‘knowledge’ from the visual to the haptic modality. However, this study leaves open the more fundamental question of whether statistical context learning is supported by uni-sensory or supra-modal memory representations. Arguably, based on Nabeta et al.’s (2003) approach, it is not possible to distinguish between these alternatives, because it is not possible to rule out that their observed haptic contextual cueing effect was visually mediated. Assuming that predictive *visual* contexts were extracted and encoded in long-term memory in the preceding visual search session, in the subsequent transfer session participants may have registered the haptically sensed configurations in a similar, *visuo-spatial* format in working memory, and it may have been this actively recoded representation (rather than a haptic representation directly) that triggered the activation of visual context information in long-term memory, thereby guiding haptic search to the target location (see also, e.g., Lederman, Klatzky, Chataway, & Summers, 1990, for a role of visual imagery in haptic recognition tasks). Given this possibility, the most direct way to address the issue of uni-sensory versus supra-modal representations mediating crossmodal transfer of contextual cueing would be to devise a multimodal search task that fosters concurrent, real-time visual-tactile interactions and so potentially the learning of the predictive crossmodal search arrays. The present study presents an attempt to implement and explore crossmodal contextual learning in such a task.

In more detail, although contextual learning of spatial target-distractor relations has been demonstrated within individual sensory modalities, mostly within vision but recently also in the domain of touch, there have been no studies that have examined for cueing across modalities, that is, from an invariant distractor context defined in one modality (e.g., touch) onto target localization in another modality (e.g., vision). The present study was designed to fill this gap in our understanding by investigating whether, and under which conditions, repeatedly encountered (invariant) tactile distractor patterns would come to facilitate search for a visual target embedded in an array of non-predictive (randomly arranged) visual distractors. For such crossmodal cueing to be possible, arguably, the underlying spatial (memory) representations would have to be coded in format, or reference system, shared by both modalities. So the critical question investigated was: is there evidence of a modality-independent (or -transcendent) representation of spatial target-distractor relations across the modalities of vision and touch?

To investigate crossmodal spatial context learning, in the current study we had observers search for a (response-relevant) visual target presented amongst homogeneous (response-irrelevant) visual and tactile non-targets, or ‘distractors’, the latter including, with regard to the target location, spatially predictive/non-predictive *tactile* distractors and spatially non-predictive *visual* distractors. Of note, the visual and tactile stimuli were presented at spatially overlapping locations in two (vertically offset) presentation planes (see Fig. 1A for an illustration), which is known to foster crossmodal learning (e.g., Shams & Seitz, 2008). The predictive tactile distractors were consistently associated with the same visual target location, thus in principle permitting the acquisition of crossmodal target-distractor (context) associations (henceforth referred to as predictive condition). By contrast, the non-predictive tactile configurations did not predict the location of the visual target (referred to as non-predictive condition). If spatial context learning operates across the modalities of vision and touch, then RTs to the visual target in predictive (tactile-context) conditions should be faster compared to responding to the visual target in non-predictive conditions. However, even a null finding would not necessarily violate the idea of supra-modal context cueing. Given the importance of vision for spatial processing (e.g., Welch & Warren, 1980; Spence, & Driver, 2004; Wesslein, Spence, & Frings, 2014), it is possible that crossmodal context cueing arises because of the remapping of the tactile stimuli into a common—visual—representation. Assuming that this remapping is a time-dependent process (e.g., Azañon & Soto-Faraco, 2008; see also Schicke & Röder, 2006), crossmodal learning of the visual target in relation to a constant tactile distractor layout might require sufficient *preview* time allowing for the positions of the tactile distractors to be processed. As detailed below, we conducted three experiments to establish the boundary conditions under which crossmodal, visual-tactile contextual cueing may be observed.

**Fig. 1:**
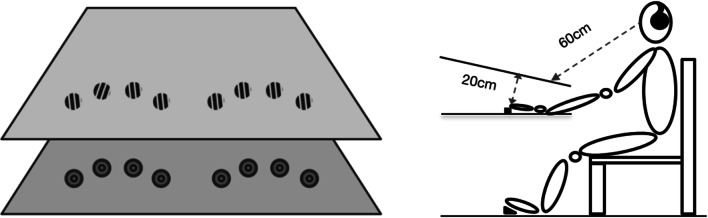
Illustration of the experimental setup. As illustrated in the left panel, tactile and visual stimuli were presented at spatially corresponding locations on a lower and an upper presentation plane, respectively. The visual stimuli consisted of eight Gabor patches subtending about 1.8° of visual angle (with a distance of about 1.9° between adjacent patches) presented, on a grey background, at eight locations positioned along two virtual ‘curves’ (one to the left and one to the right) over the horizontal axis, overlapping with the locations of the eight actuators (with a diameter of 1.8 cm, Dancer Design) on the lower plane. The target Gabor was defined by an orientation difference relative to the distractor Gabors (in the example, the target Gabor is tilted to the right compared to the distractors). As illustrated in the right panel, the height difference between the visual and tactile presentation planes was about 20 cm. Visual stimuli were presented on a white canvas surface tilted about 20° towards the observer. The viewing distance was 60 cm. Participants placed their fingers (except the thumbs) on the eight solenoid actuators

## Experiment 1

Experiment 1 was designed to examine whether the location of a *visual* target, presented in a spatially non-predictive display of visual distractors, may be learned in relation to a spatially predictive array of *tactile* distractors. Participants searched for and responded to a visual target Gabor patch with a different orientation relative to seven non-predictive distractor Gabor patches of the same (homogeneous) orientation (see Fig. 1a, b); that is, the target was a visual singleton, with the orientation contrast between the target and distractor Gabors affording ‘pop-out’ (see Liesefeld, Moran, Usher, Müller, & Zehetleitner, 2016). The visual stimuli were accompanied by the presentation of tactile items, which were delivered to participants’ fingers, with their hands placed below the visual presentation plane (see Fig. 1a). In more detail, two fingers of each hand were stimulated on every trial; exactly which ones were varied pseudo-randomly across trials and, in the predictive condition, provided the context cue for the location of the visual target (see also Assumpção et al., 2015, 2018). Half of these tactile distractor configurations were paired with the same visual target locations across trials (predictive condition), and the other half with randomly varying target locations (non-predictive condition). Accordingly, only in the predictive condition could observers eventually form unique fixed spatial associations between the visual target location and the tactile distractor configuration. If participants can develop spatial associative memory across the two modalities, RTs should become faster under conditions of predictive (constant) versus non-predictive (variable) placement of the visual target in relation to the tactile distractor arrangement.

### Method

#### Participants

Twelve right-handed volunteers (five males), with normal or corrected-to-normal vision participated in Experiment 1 (mean age = 26.25 years, SD = 6.58 years), for the payment of 9 euros per hour. All observers self-reported normal tactile sensitivity and were naive as to the purpose of the study. All provided written informed consent prior to the experiment. The experimental procedure was approved by the Ethics Committee of the LMU Munich Faculty of Psychology and Pedagogics. The sample size was determined based on previous, visual and tactile cueing studies (e.g., Chun & Jiang, 1998, 1999; Assumpção et al., 2015; Zellin et al., 2014; Zinchenko, Conci, Müller, & Geyer, 2018), aiming for 85% power to detect a relatively large effect size (f(U) = 0.8) in a repeated-measures analysis of variance (ANOVA; *η*_*p*_^*2*^ = 0.4) with an alpha level of .05. Power estimates were computed using G*Power (Erdfelder, Faul, & Buchner, 1996).

#### Apparatus and Stimuli

The experiment (conducted in a sound-attenuated testing chamber that was dimly lit by indirect incandescent lighting) was run on a Windows computer using Matlab routines and Psychophysics Toolbox extensions (Brainard, 1997; Pelli, 1997). The tactile and visual items were presented at spatially corresponding locations on a lower (tactile) and upper (visual) presentation plane. Visual stimuli (and task instructions/ feedback) were projected onto a white canvas in front of the participant, using an Optoma projector (HD131Xe; screen resolution: 1024 × 720 pixels; refresh rate: 60 Hz). The projector was mounted on the ceiling of the experimental booth behind the participants. The canvas was fixed on a wooden frame, whose back was tilted about 20° towards the observer. The viewing distance was fixed at about 60 cm (see Fig. 1). Tactile stimuli were delivered via vibrotactile stimulators (solenoid actuators with a diameter of 1.8 cm, Dancer Design). The actuators activated lodged metal tips vibrating a pin 2–3 mm following the magnetization of the solenoid coils, controlled by a 10-channel Tactor Amplifier (Dancer Design) connected to the computer with a MOTU analog output card.

The visual stimuli consisted of eight Gabor patches (Michelson contrast 0.96, the spatial frequency of 2 cpd), each subtending about 1.8° of visual angle, presented on a grey background (mean luminance of 36.4 cd/m^2^). One patch was the (visual) target and seven were homogeneous visual distractors. These stimuli were presented at eight locations positioned along two virtual ‘curves’ (one to the left and one to the right) over the horizontal axis, with four locations per curve; these curves were meant to replicate the curvature formed by the locations assigned to each of the four actuators at each hand (see Fig. 1). The distance between adjacent items was set at about 1.9° of visual angle. The tilt degree of distractor Gabor patches (i.e., X_d_°) was –2°. The target orientation was defined by X_d_° ± ΔX° (i.e., tilted to the left/right compared to the distractors). ΔX° was set to 7.2°, based on a pilot experiment (unpublished data) designed to make the mean search time for the visual target (presented amongst visual distractors) comparable to that required to detect a tactile target amongst tactile distractors, that is, to make the visual search task comparable, in terms of difficulty, to the tactile task.

In each display, in addition to the eight visual items (one target, seven distractors), four tactile distractors were presented simultaneously. Participants placed their fingers (except the thumbs) on eight solenoids delivering identical tactile stimulation (see Assumpção et al., 2015, for details). The four vibrotactile stimulations were delivered to two (selected) fingers of each hand via the solenoid actuators. The exact locations of the actuators were set corresponding to the locations of the visual stimuli, though being individually adjusted at the same time for participants’ optimal comfort and performance (i.e., with a distance of about 2 cm between adjacent actuators, but varying within ~ 0.5 cm in the Y- and X-directions). The solenoid actuators vibrated constantly at 150 Hz until a response was issued or the trial was timed out. During the experiment, participants were asked to wear headphones (Philips SHL4000, 30-mm speaker drive), through which white noise (65 dBA) was delivered to mask the tactile vibrations that would otherwise have been audible in the sound-insulated testing cabin (see also Assumpção et al., 2015, 2018).

#### Paradigm and Procedure

**Fig. 2:**
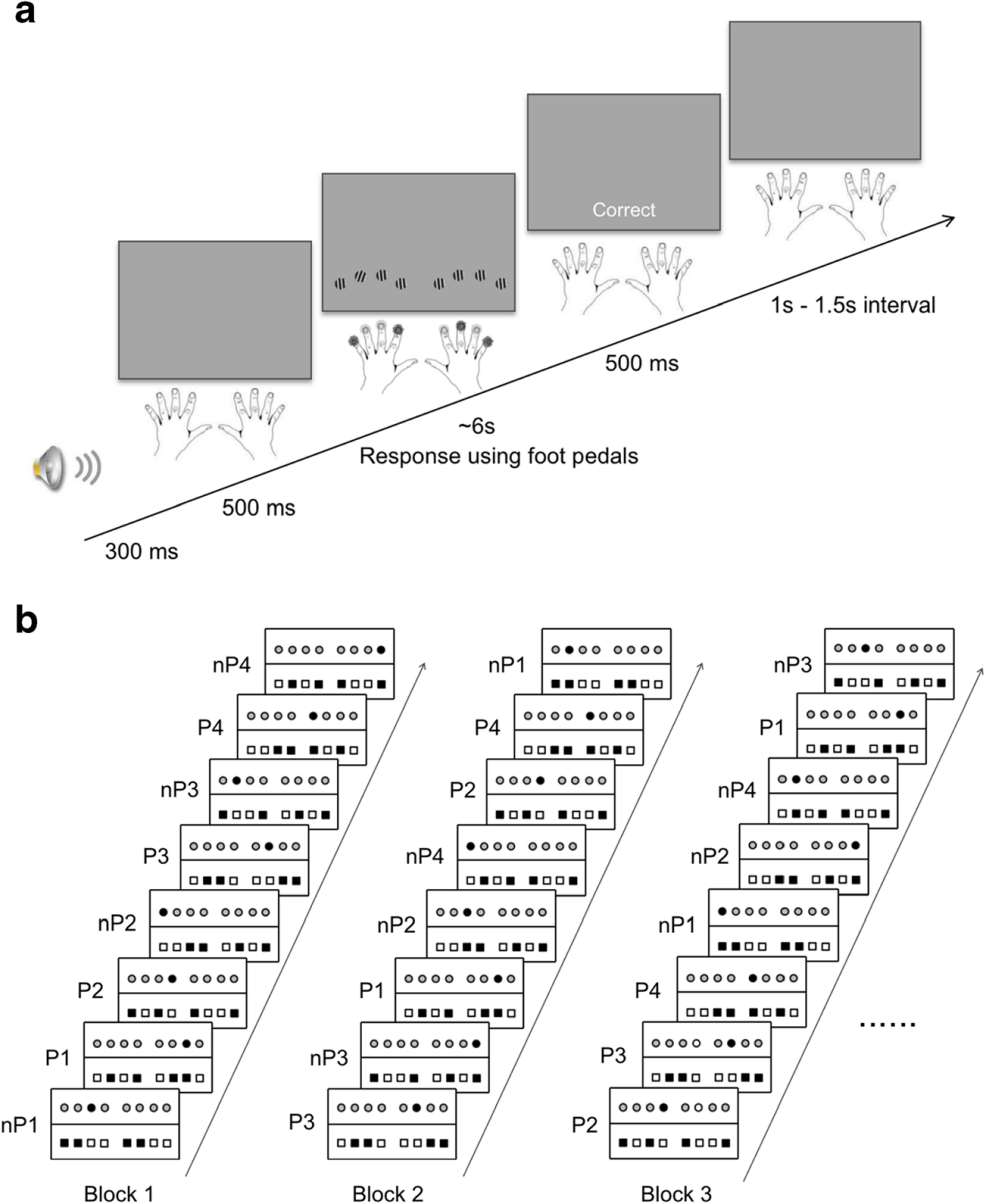
**a** An example trial sequence of the visual-tactile search task. Tactile and visual stimuli were presented at the same time after the initial beep signal. *Dark circles* represent the stimulated fingers, and *light grey circles* the non-stimulated fingers. Observers’ task was to respond to the orientation of the visual target via corresponding foot pedals (with target-pedal assignment counterbalanced across participants). A feedback display was presented after the response. **b** A possible sequence of three blocks. Two sets of four configurations (predictive and non-predictive) were randomly generated for each participant. Four of the visual target locations were assigned to predictive (P) configurations, and the remaining four target locations were assigned to non-predictive (nP) configurations. Across participants, each of the target locations was assigned equally often to P and nP configurations. In P configurations, the positions of the tactile distractors (*filled squares*) were associated with the same visual target location (*filled circles*) across blocks. In nP configurations, although the positions of the tactile distractors were constant, the visual target was presented at variable locations across trials/ blocks. The sequence of P and nP trials was randomized within each block

Each trial began with a beep (600 Hz) for 300 ms to indicate the start of the trial. After a short interval of 500 ms, actuators began to vibrate at the same time as the onset of the visual search array. The visual target was defined randomly as left- or right-tilted Gabor patch relative to the distractor orientation. Participants had to respond to the orientation of the visual target Gabor patch as quickly and accurately as possible. During the instruction phase, they were told to attend to the tactile as well as the visual stimuli. Responses were recorded using foot pedals (Heijo Research Electronics, UK). For example, when the tilt of the target was left (right), the participant had to press the left (right) foot pedal. Target-pedal assignment was counterbalanced across participants. Visual and tactile stimuli were presented until a response was executed or until a maximum duration of 6000 ms had elapsed. Next, a feedback screen (indicating “correct” or “wrong” response) was presented centrally for 500 ms. After an inter-trial interval of 1000 to 1500 ms, the next trial began (see Fig. 2a). At the end of each block, information about ‌the‌ ‌mean‌ ‌accuracy‌ ‌attained‌ ‌in‌ ‌that‌ ‌search‌ ‌block was provided in the center of the screen for 1000 ms.

Two sets of four configurations (predictive and non-predictive) were randomly generated for each participant (see Fig. 2b). In the predictive condition, four of the eight possible visual target locations were associated with each individual predictive tactile configuration, and this one-to-one pairing was held constant throughout the experiment. In the non-predictive condition, the other set of (constant) tactile distractor layouts did not predict the visual target location. Rather, each of the remaining four visual target positions could be paired randomly with each of the four individual tactile configurations, so that the visual target positions were not associated with any tactile configurations across blocks (see, e.g., Chaumon, Drouet, & Tallon-Baudry, 2008; Chaumon, Schwartz, & Tallon-Baudry, 2009 for this approach). The experiment was divided into 50 blocks of eight trials each (without gaps between the blocks), with four predictive and four non-predictive configurations. To rule out learning effects such as those that could eventually arise from a constant (repeated) sequence of target positions in previous blocks of eight trials on search performance in a given, current block, predictive and non-predictive layouts and target locations within these layouts were presented on occasions (trials) that were determined randomly within each eight-trial block. Further, across participants, each of the eight target locations was assigned equally often to predictive and non-predictive configurations so as to (1) exclude target location repetition effects (i.e., probability cueing; see, e.g., Geng & Behrmann, 2005); and (2) target location distance effects, that is, potentially confounding effects arising from more central versus more peripheral positioning of target locations and, associated with this, differences in overall search speed (e.g., Wolfe, O’Neill, & Bennett, 1998) across predictive and non-predictive displays.

Participants were not informed in any way of the aims of the experiment. Following written and verbal instructions, each observer was familiarized with the experimental setup. They then started three practice blocks of a total of 24 trials. After each trial of the first two practice blocks, participants were asked to (explicitly) report which four fingers had been stimulated, to make sure that they carefully attended to the locations of tactile stimuli. Participants went on to perform the main experimental task only if they achieved an accuracy level > 85%. Otherwise, participants were required to repeat the practice trials. After the experiment, participants were first asked to report anything they had noticed about the experimental task, whereupon they were administered an explicit (yes–no) recognition test in which they had to indicate whether they had already perceived a given display layout—consisting of the visual target, the tactile distractors, and the visual distractors—during the prior search experiment. In this recognition test, half of the trial displays included predictive tactile configurations from the previous search task, and the other half newly generated configurations not presented before.

Error trials and trials with extreme RTs (below 200 ms or 2.5 standard deviations, SD, above individuals’ condition means) were excluded from RT analysis. To increase statistical power in the assessment of contextual-cueing effects, the data were averaged across ten consecutive blocks, which yielded five experimental epochs (of 80 trials each). Performance accuracy and RTs were then calculated per configuration and epoch for each participant and submitted to a repeated-measures ANOVA with the factors Configuration (predictive vs. non-predictive) and Epoch (1–5). A polynomial contrast was applied to the factor Epoch. For non-significant effects, we additionally report Bayes factors (*BF*_*10*_; see Jeffreys, 1961; Kass & Raftery, 1995), obtained with JASP (Love et al., 2015). A Bonferroni correction was applied in case multiple comparisons were conducted.

## Results

### Accuracy

Overall accuracy averaged 93.3%. A repeated-measures ANOVA with the factors Configuration (predictive vs. non-predictive) and Epoch (1–5) revealed the main effect of Epoch to be significant, *F*(4, 44) = 3.74, *p* = .011, *η*_*p*_^*2*^ = .25, reflecting a linear increase in accuracy as the experiment progressed (90, 93, 93, 95, 94% for Epochs 1 to 5; *F*(1, 11) = 5.25, *p* = .04, *η*_*p*_^*2*^ = .32). No other effects were significant (all *p* > .36, *η*_*p*_^*2*^ < .08, *BF*_*10*_ < .49).

### RT performance

**Fig. 3:**
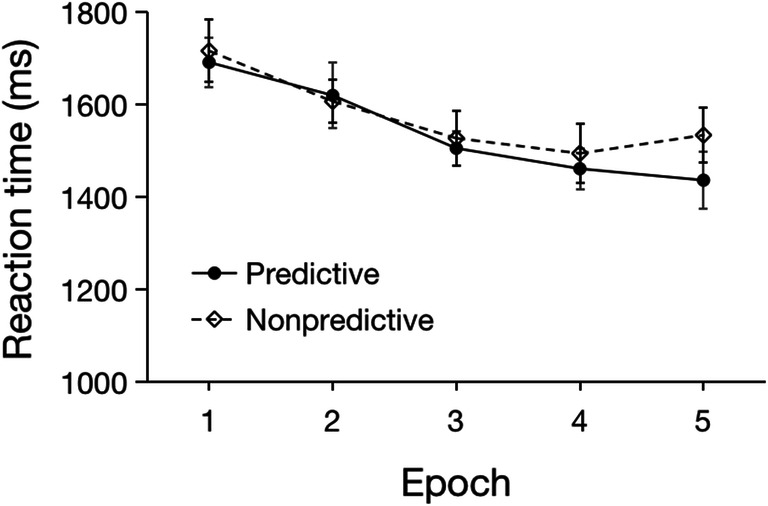
Mean RTs as a function of Epoch, separately for predictive and non-predictive configurations in Experiment 1 (one experimental ‘epoch’ summarizes the RT data across a set of ten consecutive blocks). The *error bars* represent the within-subject standard error of the mean. The *dashed line* denotes the non-predictive condition, the *solid line* the predictive condition

Extreme RTs occurred in 4% of all trials. The mean RT of the valid trials (i.e., excluding error trials and trials with extreme RTs) was 1560 ms (SE = 123 ms). Figure 3 depicts the (group) mean RTs for predictive and non-predictive configurations as a function of epoch. A repeated-measures ANOVA of individuals’ mean RTs with the factors Configuration (predictive vs. non-predictive) and Epoch (1–5) only revealed a significant main effect of Epoch, *F*(4, 44) = 4.06, *p* = .007, *η*_*p*_^*2*^ = .27: there was a linear decrease in RTs as the experiment progressed (1704, 1614, 1517, 1478, 1486 ms for Epochs 1 to 5; *F*(1, 11) = 7.33, *p* = .02, *η*_*p*_^*2*^ = .40). Neither the main effect of Configuration (predictive: RT = 1544 ms, SE = 58 ms; non-predictive: RT = 1576 ms, SE = 62 ms), *F*(1, 11) = .27, *p* = .61, *η*_*p*_^*2*^ = .02, *BF*_*10*_ = .25, nor the Configuration × Epoch interaction, *F*(4, 44) = .71, *p* = .59, *η*_*p*_^*2*^= .06, *BF*_*10*_ = .08, were significant.

### Recognition performance

Participants’ explicit recognition performance – that is, their ability to tell apart predictive tactile displays (‘signals’) from non-predictive displays (‘noise’) – was assessed by the signal-detection sensitivity parameter *d’* (Green & Swets, 1966), treating correct recognition of predictive displays as ‘hit’ responses and incorrect ‘recognition’ of non-predictive displays as ‘false-alarm’ responses. Across participants, *d’* was relatively small (– 0.28) and statistically indistinguishable from zero, *t*(11) = – 1.51, *p* =.16, *d* = – 0.44, *BF*_*10*_ = 0.71, providing little evidence that participants consciously recognized the predictive displays. Moreover, none of the participants spontaneously reported having noticed the display repetition during the search task.

## Discussion

Experiment 1 examined for the presence of contextual cueing in a novel, multimodal search environment in which a constant location of a visual target was predicted, or potentially ‘cued’, by constant distractor locations in the tactile (rather than, as is typical in contextual-cueing paradigms, the visual) modality. The results revealed a main effect of Epoch for both performance accuracy and speed, indicative of increasingly efficient task execution as the experiment progressed, reflecting procedural learning (e.g., Schneider & Shiffrin, 1977). Critically, however, we did not observe any RT advantage for predictive (tactile distractors predict the location of the visual target) over non-predictive distractor arrays (tactile distractors bear no consistent relation to the location of the visual target). This outcome suggests that, at least under the conditions of Experiment 1, observers are unable to form spatial context associations between the location of the visual target and the predictive tactile configuration.

Recent evidence suggests that tactile events can be represented relative to where they occur on the (anatomical) body surface (e.g., Medina, McCloskey, Coslett, & Rapp, 2014; Kuroki, Watanabe, Kawakami, Tachi, & Nishida, 2010) or relative to the position of the body (limbs) in external space (e.g., Driver & Spence, 1998; Kennett et al., 2002; Azañón & Soto-Faraco, 2008; Azañón, Stenner, Cardini, & Haggard, 2015; Schicke & Roder, 2006). Furthermore, it has been shown that localizing touch to a finger involves the transformation of the initially sensed anatomical skin-surface location to external finger coordinates (e.g., Badde, Röder, & Heed, 2014, 2015). However, Badde and collaborators also showed that tactile remapping is not an ‘obligatory’ process in that the use of one (anatomical) over the other (external) frame is flexible, depending on the specific circumstances of the task (Badde et al., 2015; Badde & Heed, 2016; Buchholz, Jensen, & Medendorp, 2011, 2013; Heed & Röder, 2010; Schubert et al., 2015). Concerning finger localization, in a recent study (Assumpção et al., 2018), the locations of the search stimuli (fingers) are learned with respect to an anatomical reference frame during tactile search. But, concerning the concurrent availability of different (somatotopic vs. external) reference frames in tactile tasks, it has also been shown that effective utilization of an external frame requires a time-consuming process of remapping the initially somatotopically sensed items (e.g., Azañón & Soto-Faraco, 2008; Kennett, Spence, & Driver, 2002). On the assumption that crossmodal contextual cueing requires a common, external frame (allowing the visual target to be associated with a configuration of tactile distractors), the beneficial effects of the consistently positioned tactile distractors in the search for a visual target may become measurable only when observers have sufficient *preview* time of the distractor arrangement, permitting the distractors’ (somatotopic) coordinates to be remapped into an external reference frame. Accordingly, insufficient preview time could explain why no crossmodal contextual cueing developed in Experiment 1.

A null result would also have been predicted by accounts simply assuming that the encoding of tactile patterns spanning multiple locations is a difficult, time-consuming process (Gallace & Spence, 2014). Thus, with the simultaneous presentation of the tactile and visual stimuli in Experiment 1, observers’ attention may have already been preferentially allocated to the task-relevant visual items (which were also made ‘salient’ by their sudden onset), leaving insufficient resources for the processing of the predictive distractor configurations. In fact, Geyer, Zehetleitner, and Müller (2010b) had shown that, in order for contextual cueing to become manifest in a visual singleton (‘pop-out’) search task (similar to the task variant employed in the present study), the predictive visual item layout had to be presented to participants, in the form of placeholder stimuli, prior to the search display in which the placeholders transmuted into the target and distractor stimuli. Thus, even in a purely intra-modal visual search task (with the target being singled out by rapid saliency computations), the context had to be previewed to have a search-guiding effect. Accordingly, to test whether a sufficiently long preview of the tactile items is a critical factor for tactile cueing of visual locations to develop, the time available to process the tactile stimuli was extended in Experiment 2.

## Experiment 2

Experiment 2 set out to investigate whether spatial context associations between the visual target and the predictive tactile configuration could be formed when the tactile configuration is available for a sufficient period of time, prior to the onset of the visual array. Previous studies showed that remapping of tactile events from the initial anatomical to a subsequent external reference frame takes some 180 to 360 ms to happen (e.g., Azañón & Soto-Faraco, 2008; but see, e.g., Overvliet, Azañón, & Soto-Faraco, 2011 or Brandes & Heed, 2015, for evidence of even shorter remapping times). Thus, to ensure ample processing time and sufficient attention to the encoding of the tactile distractor patterns, tactile stimuli were presented 700 ms prior to the visual stimuli in Experiment 2. The question was: would the predictive tactile configurations come to provide an informative spatial context for the location of the visual target under these conditions?

### Method

The method of Experiment 2 was identical to Experiment 1, except that the tactile stimuli were presented 700 ms before the onset of the visual stimuli, and the stimulation continued until either a response was executed or a maximum duration of 6000 ms was reached (see Fig. 2a). A new group of 12 right-handed volunteers (five males; mean age = 23.75 years, SD = 3.02 years) was tested. All participants had normal or corrected-to-normal vision and were naive as to the purpose of the experiment. The experiment started with 24 practice trials, followed by 50 experimental blocks of eight trials each (identical to Experiment 1).

### Results

#### Accuracy

Overall accuracy was 93%. A repeated-measures ANOVA with the factors Configuration (predictive vs. non-predictive) and Epoch (1–5) on performance correct yielded a marginally significant main effect of Epoch, *F*(4, 44) = 2.25, *p* = .08, *η*_*p*_^*2*^ = .17 (with a marginal linear increase in accuracy with Epoch: 91, 93, 93, 93, 95% for Epochs 1 to 5, *F*(1, 11) = 4.04, *p* = .07, *η*_*p*_^*2*^ = .27). None of the other effects was significant (all *p* > .23, *η*_*p*_^*2*^ < .12, *BF*_*10*_ < .44). Of note, there was no significant difference between Experiments 1 and 2 in performance accuracy, *t*(22) = 0.19, *p* = 1, *d* = 0.07, *BF*_*10*_ = 0.38.

#### RT performance

**Fig. 4:**
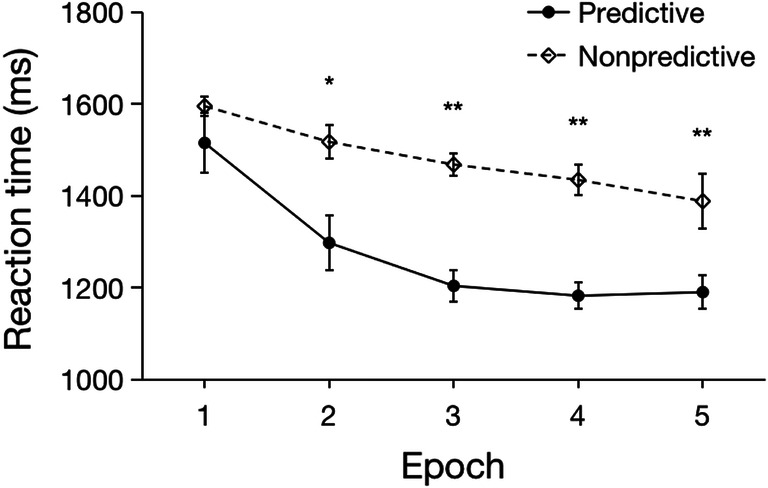
Mean RTs as a function of Epoch, separately for the predictive and non-predictive configurations in Experiment 2 (one experimental ‘epoch’ summarizes the RT data across a set of ten consecutive blocks of eight trials each). The *error bars* represent the within-subject standard error of the mean. The *dashed line* denotes the non-predictive condition, the *solid line* the predictive condition. *Asterisks* represent significance levels of *p* < .01 (**) and *p* < .05 (*)

Outlier RTs occurred in 3% of all trials, which (together with incorrect-response trials) were excluded from the RT analysis. The mean RT of the valid trials was 1380 ms (SE = 107 ms). Figure 4 presents the RTs results. A repeated-measures ANOVA with the factors Configuration (predictive vs. non-predictive) and Epoch (1–5) again revealed a significant main effect of Epoch, *F*(4, 44) = 11.03, *p* < .0001, *η*_*p*_^*2*^ = .50, with a linear decrease in RTs as the experiment progressed (1555, 1408, 1336, 1309, and 1290 ms, for Epochs 1 to 5; *F*(1, 11) = 14.46, *p* = .003, *η*_*p*_^*2*^ = .57). Importantly, this time, the main effect of Configuration, *F*(1, 11) = 17.23, *p* = .002, *η*_*p*_^*2*^ = .61, and the Configuration × Epoch interaction, *F*(4, 44) = 3.11, *p* = .02, *η*_*p*_^*2*^ = .22, also turned out significant. The main effect of Configuration was due to participants responding overall faster to targets with predictive (RT = 1278 ms, SE = 106 ms) as compared to non-predictive tactile configurations (RT = 1481 ms, SE = 114 ms). And the interaction reflected the fact that the RT advantage for predictive over non-predictive configurations became reliable from Epoch 2 onwards (Epoch 2: *p* = .02, *d* = 0.78; Epoch 3: *p* < .001, *d* = 1.66; Epoch 4: *p* < .001, *d* = 1.46; Epoch 5: *p* = .002, *d* = 1.18; see Fig. 4 for the development of the cueing effect across epochs), with no advantage in Epoch 1 (Epoch 1: *p* = .29, *d* = .32, *BF*_*10*_ = .48). As with response accuracy (see analysis above), there was no significant difference between Experiments 1 and 2 in terms of the overall RT level, *t*(22) = 1.10, *p* =.28, *d* = 0.45, *BF*_*10*_ = 0.58.

#### Recognition performance

Overall, *d’* was quite small (-0.17) and statistically indistinguishable from zero, *t*(11) = – 0.91, *p* =.38, *d* = – 0.26, *BF*_*10*_ = 0.41. No subject reported awareness of display repetitions during the search task.

## Discussion

Experiment 2 revealed context-independent procedural learning as the experiment progressed, as already seen in Experiment 1. However, unlike Experiment 1, with a 700-ms preview time of the tactile configuration, RTs to the visual target became faster with predictive, as compared to non-predictive, tactile displays, thus positively demonstrating observers’ ability to form spatial context associations across the modalities of vision and touch; furthermore, the crossmodal cueing effect was sustained as the experiment progressed. Thus, Experiment 2 shows that, given sufficient encoding time of the predictive tactile distractor configuration, spatial context associations can be extracted and used to guide focal attention in multisensory search arrays. Next, we examined whether the acquired context memory represents patterns in a format common to both modalities, which would require a process of remapping from the initially somatotopically sensed tactile items into a common, external frame.

## Experiment 3

Experiments 1 and 2 show that the learning and retrieval of crossmodal spatial (visual) target to (tactile) context associations requires additional time for the processing of the tactile configuration, ahead of the presentation of the visual target. One possible reason (amongst others) for this might have to do with the format of the spatial memory representation mediating the crossmodal search-guidance effect: the tactile configuration may have to be remapped from an initially somatotopic into an external reference frame, that is, a common representational format for both the visual and tactile items. Given this, Experiment 3 was designed to probe the (somatotopic vs. external) reference frame of the representation affording visual-tactile contextual cueing, by introducing a hand-posture manipulation along the lines of Assumpção et al. (2018). For the first four epochs of the experiment, participants performed the crossmodal search for a visual target with their hands stretched out in a parallel position, but later on, in Epoch 5, they were required to flip their hands up (down) by 180° relative to the preceding four epochs. Under these ‘flipped-hand’ conditions, the tactile distractors in the predictive condition stayed at the same fingers (so somatotopy was preserved), but they now appeared at different external coordinates. Prior research with a unimodal tactile search task had shown that tactile contextual cueing remains intact with this manipulation (i.e., despite the flipping of the hands), suggesting that cueing is based on somatotopic, finger-centered representations (Assumpção et al., 2018). However, if, in the crossmodal visual-tactile search task implemented in the present study, the tactile distractors undergo remapping from a somatotopic to an external frame, the change (flipping) of the hand positions in Epoch 5 should strongly affect the (initially, in Epochs 1–4, acquired) contextual cueing effect – as, after the change, the locations of the tactile items differ in external space (even though the very same fingers would still be stimulated). Thus, the remapping hypothesis (but not the somatotopic hypothesis) predicts the abolishment of crossmodal contextual cueing by the change of the hand position.

### Method

Experiment 3 was a replication of Experiment 2, except that during Epoch 5 (i.e., the last ten blocks), participants flipped their hands up (down) by 180° relative to the preceding epochs (with pre-/post-position of the hands counterbalanced across participants). The flipping of the hands produced a change in the external locations of the tactile distractors, while keeping their somatotopic (finger) positions constant. A new group of 12 right-handed volunteers (six males; mean age = 23.50 years, SD = 3.12 years) was tested. All participants had normal or corrected-to-normal vision and were naive as to the purpose of the experiment. The experiment started with 24 practice trials, followed by 50 experimental blocks of eight trials each.

## Results

### Accuracy

Overall accuracy was 95.8%. Trials with the same settings as in Experiment 2 (i.e., trials from epochs 1–4, prior to the flipping of the hands in Epoch 5) were analyzed by a two-way repeated measures ANOVA with the factors Configuration (predictive, non-predictive) and Epoch (1–4). For the accuracy scores, all (main and interaction) effects were non-significant (all *p* > .19, *η*_*p*_^*2*^ < .15, *BF*_*10*_ < .97). There was also no indication of a difference between predictive (96%) and non-predictive (96%) configurations in Epoch 5, in which the hands were flipped, *t*(11) = .15, *p* = .88, *d* = .04, *BF*_*10*_ = .29.

A one-way ANOVA comparing mean response accuracy with the previous two experiments hinted at ‘marginally’ better performance in Experiment 3, *F*(2, 33) = 3.29, *p* = .05, *η*_*p*_^*2*^ = .17, *BF*_*10*_ = 1.60; however, none of the individual comparisons was significant (Experiment 3 vs. Experiment 1: *t*(22) = 2.12, *p* = .13, *d* = 0.88, *BF*_*10*_ = 1.89; Experiment 3 vs. Experiment 2: *t*(22) = 2.31, *p* = .08, *d* = 1, *BF*_*10*_ = 3.18).

### RT performance

**Fig. 5:**
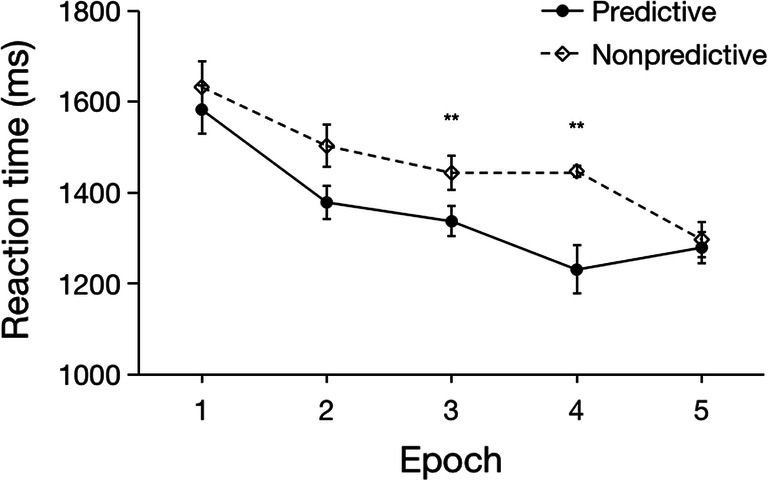
Mean RTs as a function of Epoch, separate for the predictive vs. non-predictive configurations in Experiment 3 (one experimental ‘epoch’ summarizes the RT data across a set of ten consecutive blocks). The *error bars* represent the within-subject standard error of the mean. The *dashed line* denotes the non-predictive condition, the *solid line* the predictive condition. *Asterisks* represent significance levels of *p* < .01 (**)

Outlier RTs occurred in 2% of all trials, which (together with incorrect-response trials) were excluded from the RT analysis. The overall RT mean on analyzed trials was 1413 ms (SE = 104 ms). Figure 5 depicts the (group) mean RTs across epochs, separately for the predictive and non-predictive condition. A repeated-measures ANOVA with the factors Configuration (predictive vs. non-predictive) and Epoch (1–4) revealed the main effect of Epoch to be significant, *F*(3, 33) = 9.80, *p* < .001, *η*_*p*_^*2*^ = .47, indicating, like in the previous experiments, a linear decrease as the experiment progressed (*F*(1, 11) = 20.95, *p* = .001, *η*_*p*_^*2*^ = .66; 1607, 1441, 1390, 1339 ms for Epochs 1 to 4). As in Experiment 2, the main effect of Configuration also turned out significant, *F*(1, 11) = 10.6, *p* = .008, *η*_*p*_^*2*^*=* .49: participants responded faster to visual targets with predictive (RT =1382 ms, SE = 109 ms) as compared to non-predictive tactile configurations (RT = 1506 ms, SE = 114 ms). The Configuration × Epoch interaction was marginally significant, *F*(3, 33) = 2.42, *p =* .08, *η*_*p*_^*2*^ = .18*.* Closer examination revealed the cueing effect (predictive vs. non-predictive configurations) to be reliable in Epochs 3 and 4 (Epoch 3: *p* = .006, *d* = .99; Epoch 4: *p* = .003, *d* = 1.12), but not in Epochs 1 and 2 (Epoch 1: *p* = .44, *d* = .23, *BF*_*10*_ = .38; Epoch 2: *p* = .07, *d* = .58, *BF*_*10*_ = 1.28); see Fig. 5 for the development of the cueing effect across epochs.

A one-way ANOVA comparing overall RT speed with the previous two experiments failed to reveal any significant difference: *F*(2, 33) = 0.73, *p* = .49, *η*_*p*_^*2*^ = .04, *BF*_*10*_ = 0.31 (Experiment 3 vs. Experiment 1, *t*(22) = – 0.93, *p* = 1, *d* = – 0.37, *BF*_*10*_ = 0.50; Experiment 3 vs. Experiment 2, *t*(22) = 0.21, *p* = 1, *d* = 0.09, *BF*_*10*_ = 0.38).

**Fig. 6:**
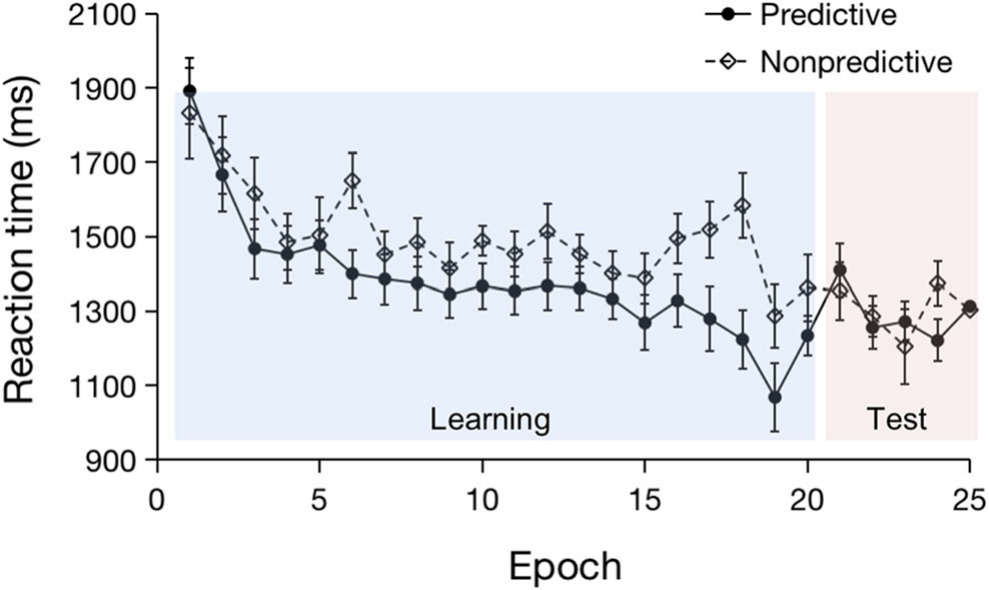
Mean RTs as a function of Epoch, separately for predictive and non-predictive configurations in Experiment 3. In this finer-grained analysis (compared to that presented in Fig. 5), pairs of two rather than ten consecutive blocks were combined into one experimental ‘epoch’, resulting in 25 epochs in total. The *error bars* represent the within-subject standard error of the mean. The *dashed line* denotes the non-predictive condition, the *solid line* the predictive condition.

Of importance with regard to the question at issue, in Experiment 3 (in contrast to Experiment 2), there was no longer a cueing effect in Epoch 5 in which the hands were flipped, *t*(11) = 0.56, *p* = .58, *d* = 0.16, *BF*_*10*_ = 0.33. Figure 5 suggests that the abolition of contextual cueing after the hand position change (in Epoch 5, which averages the data across ten consecutive blocks) was, at least in part, owing to responses being expedited with non-predictive displays. However, a finer-grained analysis, with an ‘Epoch’ averaging the data across every two consecutive blocks (thus dividing the whole experiment into 25 epochs), revealed that the decrease in response speed with non-predictive displays actually occurred prior, and so was unrelated, to the hand-posture manipulation (see Fig. 6): RTs to non-predictive displays did not differ significantly between Epoch 20 (the last ‘learning’ epoch with the original hand position) and Epoch 21 (the first ‘test’ epoch with the flipped position), *t*(11) = 0.08, *p* = .94, *d* = 0.02, *BF*_*10*_ = .29. For the predictive condition, by contrast, the difference in RTs between ‘learning’ Epoch 20 and ‘test’ Epoch 21 was significant, *t*(11) = – 2.25, *p* = .046, *d* = – 0.65. That is, there was a reliable increase in RTs to predictive displays when participants swapped their hands at the transition from the learning to the test phase. By contrast, no such effect was observed for non-predictive displays, in which RTs remained essentially stable across the final learning and the first test epoch (i.e., Epochs 20 to 21). This pattern rules out that the observed RT variation (increase) is attributable to the motor action of the hand posture change as such (e.g., Tomassini, Gori, Burr, Sandini, & Morrone, 2012), in which case one would have expected RTs to increase not only for predictive, but also for non-predictive arrays. Accordingly, the fact that an RT increase was observed only for predictive displays suggests instead that it is a consequence of the removal of learned contextual cues due to the hand posture change in the predictive condition.

### Recognition performance

Overall, *d’* was quite small (– 0.16) and statistically indistinguishable from zero, *t*(11) = – 0.75, *p* = .47, *d* = – 0.22, *BF*_*10*_ = 0.37. Again, no participant reported having noticed any display repetitions during the search task.

## Discussion

Experiment 3 replicated the crossmodal contextual-cueing effect obtained in Experiment 2. Importantly, contextual cueing was evident until observers were required to flip their hands in the last epoch (Epoch 5) of the search task. That is: contextual facilitation of search RTs disappeared when the predictive tactile distractors were presented at different external (visuo-spatial) coordinates, even though their somatotopic (finger) positions were exactly the same as before the hand-posture changed. Accordingly, our results support the idea that, in order to produce contextual cueing of visual targets, the tactile distractors must be (re-)coded in an external (visuo-spatial) reference frame. This re-coding process may well contribute to the time by which the tactile configuration must be presented ahead of the visual target in order to produce a crossmodal cueing effect, given that re-mapping of tactile events from their initial anatomical to an external reference frame is thought to take some 180 to 360 ms (e.g., Azañón & Soto-Faraco, 2008), though possibly also less time (e.g., Overvliet, Azañón, & Soto-Faraco, 2011; Brandes & Heed, 2015). Whether and to what extent the re-coding time contributes to the extra time in our paradigm will have to be assessed in future work.

## General discussion

The present study investigated whether and, if so, under which conditions spatial target-distractor relations are encoded into context memory and come to guide visual search in a multimodal search scenario, in which the target and the predictive distractors were defined in the visual and the tactile modality, respectively. The results, from three experiments, showed a consistent effect of Epoch on RTs, indicative of progressive procedural, or skill, learning as a function of the time on the task. Importantly, there was no evidence of contextual learning when the visual and tactile stimuli were presented simultaneously (Experiment 1). However, with sufficient exposure duration of the tactile items ahead of the visual array, context-based facilitation of RTs emerged (after one or two epoch of practice) when the locations, that is, the configuration, of the tactile distractors were predictive of the visual target location (Experiments 2 and 3). Apart from replicating this crossmodal contextual-cueing effect, Experiment 3 indicated that search-guiding context memories are successfully established only when the (initial) somatotopic representation of the tactile configurations can be translated into an external reference system shared with the visual modality. Furthermore, post-experimental recognition tests revealed participants’ ability to distinguish predictive (old) from non-predictive (new) conditions only to be at chance level – even in Experiments 2 and 3 in which reliable crossmodal contextual facilitation of the search RTs did develop. Taken together, the results show that spatial context associations can be successfully formed across the sensory modalities of vision and touch, but linking a given tactile configuration with the location of a visual target requires the positions of the tactile items to be remapped into external, visuo-spatial coordinates.

To our knowledge, the present findings are the first to show that visual search can be facilitated by a spatial configuration of distractors formed in a different modality, in line with the idea that there is communication between visual and tactile configural representations (Nabeta et al., 2003). In the current study, the target was defined in the visual modality, while the predictive distractors were defined in the tactile modality. The finding of a reliable cueing effect indicates that there can indeed be crossmodal learning (rather than just crossmodal transfer, as demonstrated by Nabeta et al.) of visual-tactile layouts. The present study thus provides new insights into spatial-context learning and the accessibility of spatial information across sensory modalities: consistent target-distractor relations across the modalities of vision and touch could be successfully extracted and used for visual search guidance in the same task scenario.

Note that in our study, participants’ hands were not visible, making it difficult for them to construct or use possible external references from the fingers’ spatial positions. Despite this, in Experiment 3, the contextual-cueing effect disappeared immediately once participants flipped their hands up (down) by 180^°^ after having acquired the effect, suggesting that the learned visual-tactile associations are represented in external coordinates. Guttman et al. (2005) demonstrated that learning with the non-dominant modality may still recruit mechanisms (representations) of the dominant modality. On the assumption that spatial learning is dominated by the visual modality, the formation of tactile-visual associations would likely be supported by the most task-appropriate, that is, an external, visuo-spatial representation. In other words, the tactile stimuli are re-coded in a common format, permitting them to be associated with the external coordinates used by the visual system. Whether this format is genuinely supra-modal (i.e., modality-independent), as opposed to inherently visual in nature may be a matter of debate. However, since the notion of a sensory-independent visual representation of the learned multimodal configurations is very hard to distinguish from that of sensory-independent, spatially abstract representations, we consider both possibilities to be instances of supra-modal context memory.

As indicated by previous studies (e.g., Assumpção et al., 2018), the primary representation of tactile information is anatomical skin-based. However, when the task is changed from ‘focusing’ on the body (purely tactile search) to the external visual environment (visual search with a tactile context) – the latter requiring accurate localization of the visual target and discrimination of its response-defining (orientation) feature – observers’ attention would, predominantly, be allocated to the visual domain. To establish a common, crossmodal reference frame in this situation, the tactile information would have to be remapped from a default, somatotopic (body-centered) into an external, visuo-spatial format for this information to be able to guide visual search (see also, e.g., Lederman, Klatzky, Chataway, & Summers, 1990). That is, spatial coding of tactile context requires a task-oriented remapping process (see also Buchholz et al., 2011, 2013; Heed & Röder, 2010; Badde & Heed, 2016). This is consistent with previous studies showing that external representations of tactile stimuli come into play only later on in the trial, after their initial registration in anatomical coordinates (e.g., Azañón & Soto-Faraco, 2008; Kennett et al., 2002).

It might be contended that the failure to find contextual cueing in Experiment 1 was simply owing to difficult, ‘cumbersome’ processing in the tactile modality (Gallace & Spence, 2014) and, given this, immediate allocation of attention to the visual stimuli (though see Geyer et al., 2010b, who found that a pre-search context preview was required for cueing to manifest even within a purely intra-visual pop-out search task). Accordingly, simply ensuring enough attention to, and processing time of, the tactile stimuli prior to the visual search array should be sufficient for crossmodal contextual cueing to emerge (which would be consistent with the results of Experiment 2). However, the results of Experiment 3 suggest that merely providing enough time for tactile processing is not sufficient: crossmodal contextual cueing was observed only when the tactile items could be remapped into common external coordinates shared with the visual items.

Given the limited evidence available with regard to crossmodal spatial learning, the current study raises a number of novel questions in this field. One interesting question concerns whether evidence for spatial remapping of touch in an external, supra-modal representation could be found using other, more direct approaches, such as having participants cross (rather than flip) their hands at the transition from the learning to the test phase. Under these conditions, remapping of touch would be indicated by sustained contextual cueing when the external coordinates of the tactile distractors are preserved while their anatomical locations are changed (a prediction derived from, e.g., Badde et al., 2014, 2015). Of note in this context, the relation between hand crossing and external coding of touch has recently been doubted (Badde, Röder, & Heed, 2019; Azañón & Longo, 2019), so that the hand flipping introduced in Experiment 3 might well have been the more appropriate manipulation. Another question concerns whether spatial regularities learned in the visual modality could also come to facilitate tactile search. We are currently addressing this issue, and positive findings would provide further evidence for the idea that spatial target-distractor associations may be represented in a crossmodal manner. A related issue important to examine concerns where and how visual-tactile associations are represented in the brain, to gain an understanding of the underlying functional and structural mechanisms of crossmodal contextual learning. For instance, are visual-tactile context cues merely stored in early cortical areas, where sensory representations are relatively short-lived and coded in ‘raw’ form, or are they more permanently available and reside in higher brain areas, such as the medial temporal lobes (e.g., Geyer, Baumgartner, Müller, & Pollmann, 2012; Preston & Gabrieli, 2008) that support relational learning of contextual associations in a modality-independent manner?

## Conclusions

The present study provides the first evidence that consistent visual-tactile relations can be (incidentally) acquired in a multimodal search environment and come to guide search for a visual target. In order to permit crossmodal contextual associations to be formed and to guide visual search, sufficient time is required for the tactile spatial configuration to be processed prior to the visual search array. Critically, this involves the remapping of the somatotopically sensed stimuli into common external coordinates shared with the visual stimuli. That is: the crossmodal contextual-cueing effect is supported by an external, most likely visuo-spatial, representation.

### Author Note

This work was supported by German Research Foundation (DFG) grants GE 1889/5-1, awarded to TG, and SH166/7-1 to ZS.

### Open Practices

The data and materials for all experiments are available at https://osf.io/nxy59/.
